# Dietary Trivalent Chromium Exposure Up-Regulates Lipid Metabolism in Coral Trout: The Evidence From Transcriptome Analysis

**DOI:** 10.3389/fphys.2021.640898

**Published:** 2021-02-25

**Authors:** Lu Wei, Yu Li, Hengzhen Ye, Juan Xiao, Christer Hogstrand, Iain Green, Zhiqiang Guo, Dong Han

**Affiliations:** ^1^State Key Laboratory of Marine Resource Utilization in South China Sea, School of Life and Pharmaceutical Sciences, College of Food Science and Engineering, Hainan University, Haikou, China; ^2^Metals Metabolism Group, School of Life Course Sciences, King’s College London, London, United Kingdom; ^3^Department of Life and Environmental Sciences, Faculty of Science and Technology, Bournemouth University, Poole, United Kingdom; ^4^State Key Laboratory of Freshwater Ecology and Biotechnology, Institute of Hydrobiology, Chinese Academy of Sciences, Wuhan, China; ^5^Southern Marine Science and Engineering Guangdong Laboratory, Zhanjiang, China

**Keywords:** RNA-sequencing, chromium, toxicity, lipid metabolism, fish

## Abstract

Diet quality greatly affects an animal’s performance and metabolism. Despite the fact that trivalent chromium [Cr(III)] is considered an essential element and is widely used in nutritional supplements for animals and humans, the potential toxicity of Cr(III) is unclear. Here, liver transcriptome sequencing was performed on coral trout (*Plectropomus leopardus*) exposed to 200 mg kg^–1^ of dietary organic Cr(III) [as chromium picolinate (CrPic)] for 8 weeks. One-hundred-and thirteen differentially expressed genes (DEGs) were identified in response to Cr(III) stress, in comparison to the control, including 31 up-regulated and 82 down-regulated DEGs. Clusters of Orthologous Groups of proteins (COG) classifies DEGs into 15 functional categories, with the predominant category being related to lipid transport and metabolism (9.73%). The Kyoto Encyclopedia of Genes and Genomes (KEGG) assigned DEGs to six major categories with robust DEGs as part of the lipid metabolism pathway (18.58%). Moreover, KEGG functional enrichment analysis showed that these DEGs are primarily related to steroid biosynthesis, terpenoid backbone biosynthesis, and steroid hormone biosynthesis pathways, of which steroid biosynthesis was the most significant pathway, and 12 key up-regulated DEGs (*dhcr7*, *dhcr24, ebp, lss, msmo1, sqle, cyp51, tm7sf2, sc5dl, fdft1, nsdhl*, and *hsd17b7*) were found for steroid biosynthesis pathways. To validate the RNA sequencing data using quantitative real-time PCR (qRT-PCR), qRT-PCR results indicate that the expression of genes encoding HMGCR, TM7SF2, TRYP2, CTRL, EBP, LSS, and CYP51 were induced, while those encoding THRSP, LCE, and MCM5 were reduced, consistent with RNA-seq results. This findings provides the first evidence that a long-term high dose of Cr(III) intake causes lipid metabolism disorder and potential toxicity in fish. Cautious health risk assessment of dietary Cr(III) intake is therefore highly recommended for the commercial and/or natural diets of aquatic animals, which has previously largely been ignored.

## Introduction

An animal’s growth performance is largely influenced by food quality, and the safety of food affects its quality. In August 2005, the Food and Drug Administration (FDA) in the United States officially approved the production of organic chromium (CrPic) (FDA, 2005). CrPic a trivalent chromium [Cr(III)] and its safety is controversial. Despite this, Cr(III) is considered an essential element and is widely used in nutritional supplements for animals and humans (Tian et al., 2013; Hamilton et al., 2018; Mayorga et al., 2018; Krol et al., 2020).

Several studies have shown that CrPic had cardioprotective effects in hyperlipidemic rats (Shafik et al., 2017), hepatoprotective activity in alloxan dosed mice (Fan et al., 2012), and antioxidative effects in rats (Marmett et al., 2018). Specifically, Cr(III) is considered to be a glucose tolerance factor (GTF) and has an increasing insulin sensitivity effect. Cr is considered to be an essential micronutrient and is linked to many processes regulated in the human body, including activation of insulin receptors by the oligopeptide chromudulin to maintain glucose homeostasis, thereby increasing insulin sensitivity and signal transduction (NIH, 2013). Cr deficiency may cause glucose intolerance, fasting hyperglycemia, increased circulating insulin, and can even impair growth (Vincent, 2000). Wang et al. demonstrated that AMP-activated protein kinase activates the increase of dietary CrPic inhibited secretion of insulin-resistant 3T3-L1 adipocytes (Wang et al., 2009). CrPic could also improve protein deposition *via* the regulation of mRNA levels of ubiquitin (Ub), insulin-like growth factor 1 receptor (IGF-1R), and insulin-like growth factor 1 (IGF-1) in rat skeletal muscle cells (Peng et al., 2010), and glucose uptake and metabolism *via* up-regulating the mRNA levels of glycogen synthase (GS), glucose transporter 4 (GLUT4), uncoupling protein-3 (UCP3), and insulin receptor (IR) in skeletal muscle cells (Qiao et al., 2009). Dietary CrPic supplementation has been found to stimulate weight loss in humans (Tian et al., 2013).

Conversely, isolated studies have reported that CrPic caused harmful effects, such as mutagenicity in a *Drosophila* model (Hepburn et al., 2003) and in mammalian cells (Andersson et al., 2007). Additionally, mitochondrial damage and apoptosis was reported in the CHO AA8 cell line (Jiang et al., 2018) and in a bacterial model (Jiang et al., 2018). Furthermore, the deleterious effects of dietary CrPic supplementation have been confirmed in rats, including oxidative DNA damage (Andersson et al., 2007), increased lipid peroxidation (Refaie et al., 2009), and decreased antioxidative enzyme activity (Shafik et al., 2017). CrPic intake was also found to lead to oxidative damage in pigs (Tan et al., 2008), and apoptotic effects in chick embryo fibroblasts (Bai et al., 2014). In humans, in some cases, dietary Cr(III) may lead to Cr(III) accumulation in the kidneys (Stoecker, 1999). These findings raise concerns about the potential toxicity and safety of CrPic as a dietary supplement.

In aquatic animals, a few studies have demonstrated that dietary CrPic supplementation could significantly improve growth performance and carbohydrate metabolism in fish, such as mirror carp (*Cyprinus idellus*; Ahmed et al., 2012), and Nile tilapia (*Oreochromis niloticus*; Li et al., 2018). However, other studies reported that dietary CrPic supplementation did not affect the health status and the growth of rainbow trout (Selcuk et al., 2010) and Nile tilapia (Pires et al., 2015). These inconsistent results might be due to differences in species, exposure time and/or the dietary CrPic concentrations used in those studies. Nevertheless, to date, the toxicity of CrPic has been rarely investigated in aquatic animals, especially in a long-term dietary CrPic exposure scenario, which has probably been occurring in animals feeding on Cr(III) enriched commercial feeds or contaminated natural diets.

In the present study, therefore, we investigated the chronic toxicity of dietary organic Cr(III) [as chromium picolinate (CrPic)] in juvenile coral trout (*Plectropomus leopardus*), a commercially and ecologically important coral fish listed in the Red List of Threatened Species as being Near Threatened (IUCN, 2004). The aim of this study was to use transcriptomics to cast light on the mechanisms of effects elicited by dietary CrPic exposure.

## Materials and Methods

### The Experimental Animals and Sampling

Juvenile coral trout (*Plectropomus leopardus)* with an initial body weight of 15.5 ± 1.8 g (mean ± *SD*, *n* = 36) were purchased from Blue Ocean Aquaculture (Lingshui, Hainan Province, China). The fish were maintained in laboratory aquaria (0.8 × 0.6 × 0.6 m) for 2 weeks to allow acclimatization (Guo et al., 2019), during which time they were fed a commercial diet twice daily (Supplementary Table 1, Hai-Tong Biological Feeds Technology Co., Ltd., Shandong Province, China). Laboratory water conditions were; total Cr concentrations <0.025 mg L^–1^, dissolved oxygen 5.5–7.4 mg L^–1^, pH 7.3–8.4, water temperature 28.5–29.7°C, and the photoperiod was 12 h.

After 2 weeks of acclimatization, the coral trout were randomly separated into two groups–the control group and dietary CrPic treatment group (CrPic). Each group had three triplicates containing 18 fish in total, with six fish in each treatment held in one tank. In the control group, the coral trout were fed a commercial diet [the total Cr concentration in the diet was 2.21 ± 0.14 mg kg^–1^, this is a baseline concentration of Cr (Supplementary Table 1)]. The CrPic group were fed the same commercial diet, but with a nominal Cr concentration of 200 mg kg^–1^ added as chromium picolinate (Sigma-Aldrich). The measured value for the Cr content of the CrPic diet was 195.67 ± 9.97 mg kg^–1^ (Supplementary Table 1). The nominal Cr concentration of 200 mg kg^–1^ was based on our preliminary experiment and previously published studies (e.g., Kim and Kang, 2016; Staniek and Wojcik, 2018; Ren et al., 2018). The commercial diet and chromium picolinate were ground and mixed thoroughly, then pellets were reformed with a diameter of 3.0 mm using a laboratory press. Pellets were then oven dried at 60°C and stored at −20°C. During the feeding experiment, the seawater in the tank was replaced by fresh filtered seawater six times per day. The concentration of total Cr was <0.045 mg L^–1^ in the water. On the 56th day of the feeding experiment, the fish were euthanized and the livers were collected and then frozen in liquid nitrogen for later preparation for transcriptome sequencing.

### Transcriptome Sequencing

The livers from the control and CrPic group (36 fish in total; *n* = 18 per group) were used for RNA sequencing, which was completed by Majorbio Bioinformatics Technology Co., Ltd. (Shanghai, China). In brief, using TRIzol reagent (Life Technologies, California, United States) total RNA was extracted from each liver sample according to the manufacturer’s instructions. We then tested RNA degradation and pollution on 1% agarose gels, and we tested the RNA extract’s purity and concentration using the 260/280 nm ratio determined by a Nanotrop 2000 (IMPLEN, CA, United States). We used 2 μg of RNA per liver sample as the input material for the transcriptome sequencing. To generate sequencing libraries, we used NEBNext Ultra RNA Library Prep Kit according to the manufacturer’s recommendations. We then sequenced libraries on an Illumina Hiseq 2500 platform and generated 150 bp paired-end reads.

### Library Data Analysis

The raw data (raw reads) of the FASTA format were first filtered based on the value of *p* < 0.05. We obtained clean reads by removing low quality or undetermined base of raw reads. Transcriptome assembly into contigs was accomplished using the Trinity program^1^. The unigenes were annotated according to the following databases: Swiss-Prot^2^, Nr/Nt (NCBI non-redundant protein/nucleotide sequences^3^), COG (Clusters of Orthologous Groups of proteins^4^), Pfam (Protein family^5^), KEGG (Kyoto Encyclopedia of Genes and Genomes^6^), and GO (Gene Ontology^7^). To obtain significant differences in the expression levels of the unigenes, we used the DESeq2, *p*-value adjusted with Hochberg’s (HB) approach (also called FDR) < 0.05 and |log_2_ (Fold Change, FC)| ≥ 1 to calculate each gene expression and to analyze differentially expressed genes (DEGs). To determine enrichment analysis, GO and KEGG enrichment analysis were doned in the DAVID bioinformatics software (version 6.8).

### Quantitative Real-Time PCR Analysis

We randomly selected 10 differentially expressed genes for qRT-PCR verification. β*-actin* was used as an internal reference gene. The primers were designed by Primer Premier 6 (Supplementary Table 2). The amplification efficiency of the tested targeted genes is shown in Supplementary Table 3. Complementary DNA (cDNA) were synthesized. The qRT-PCR reaction was run on a QuantStudio 6 Flex Real-Time PCR System (Thermo Fisher Scientific, United States), using 2× SYBR Green qPCR Mix (Vazyme, Biotech, China) as recommended by the manufacturer’s protocol. A melting curve was generated for each qRT-PCR product. The relative mRNA expression levels of the selected genes were calculated using the 2^–Δ^ ^Δ^ ^*CT*^ method (Pfaffl, 2001).

### Statistical Analysis of mRNA Data

Statistical analyses (*t*-test and one-way ANOVA) were performed to test the difference in the mean values between different groups using SPSS 19.0 software (SPSS Inc., Chicago, IL, United States). The Shapiro-Wilk test and Levene’s test were used to verify normality and homoscedasticity of the data, respectively. All the data was presented as mean ± standard deviation (*SD*, *n* = 6).

## Results

### RNA-Seq

We obtained 62,923,240 and 56,630,392 mean raw reads in the control and CrPic group, respectively (Table 1). By removing reads from the undetermined base, containing adapter reads and low-quality raw reads, we obtained clean reads. Following this, there were 62,062,992 and 55,821,019 mean clean reads in the control and CrPic group, respectively. The obtained Q20 and Q30 were more than 94%, and the error rate was less than 0.05%. The GC content was ca. 50% in the control and CrPic-treated groups, indicating high quality transcriptome sequencing in our study.

**TABLE 1:** The statistics obtain for transcriptome sequencing of coral trout (*Plectropomus leopardus*) livers from control fish and fish exposed to 200 mg kg^–1^ dietary chromium picolinate for 8 weeks.

Liver samples	Raw reads	Clean reads	Raw bases	Clean bases	Q20 (%)	Q30 (%)	GC (%)	Error rate (%)
Con_1	62,647,874	61,738,880	9,459,828,974	9,172,460,389	98.31	94.63	51.67	0.0244
Con_2	62,868,628	62,072,640	9,493,162,828	9,229,499,398	98.31	94.63	51.07	0.0244
Con_3	63,253,218	62,377,456	9,551,235,918	9,290,266,604	98.40	94.82	50.88	0.0242
CrPic_1	58,869,468	58,123,440	8,889,289,668	8,665,129,602	98.28	94.52	50.65	0.0245
CrPic_2	61,859,500	60,935,426	9,340,784,500	9,077,530,160	98.32	94.62	50.95	0.0244
CrPic_3	49,162,208	48,404,192	7,423,493,408	7,206,155,015	98.15	94.25	50.49	0.0248

*The control groups were Con_1, Con_2 and Con_3. The treatment groups were CrPic_1, CrPic_2 and CrPic_3.*

### The Differential Expression Genes (DEGs)

**FIGURE 1:**
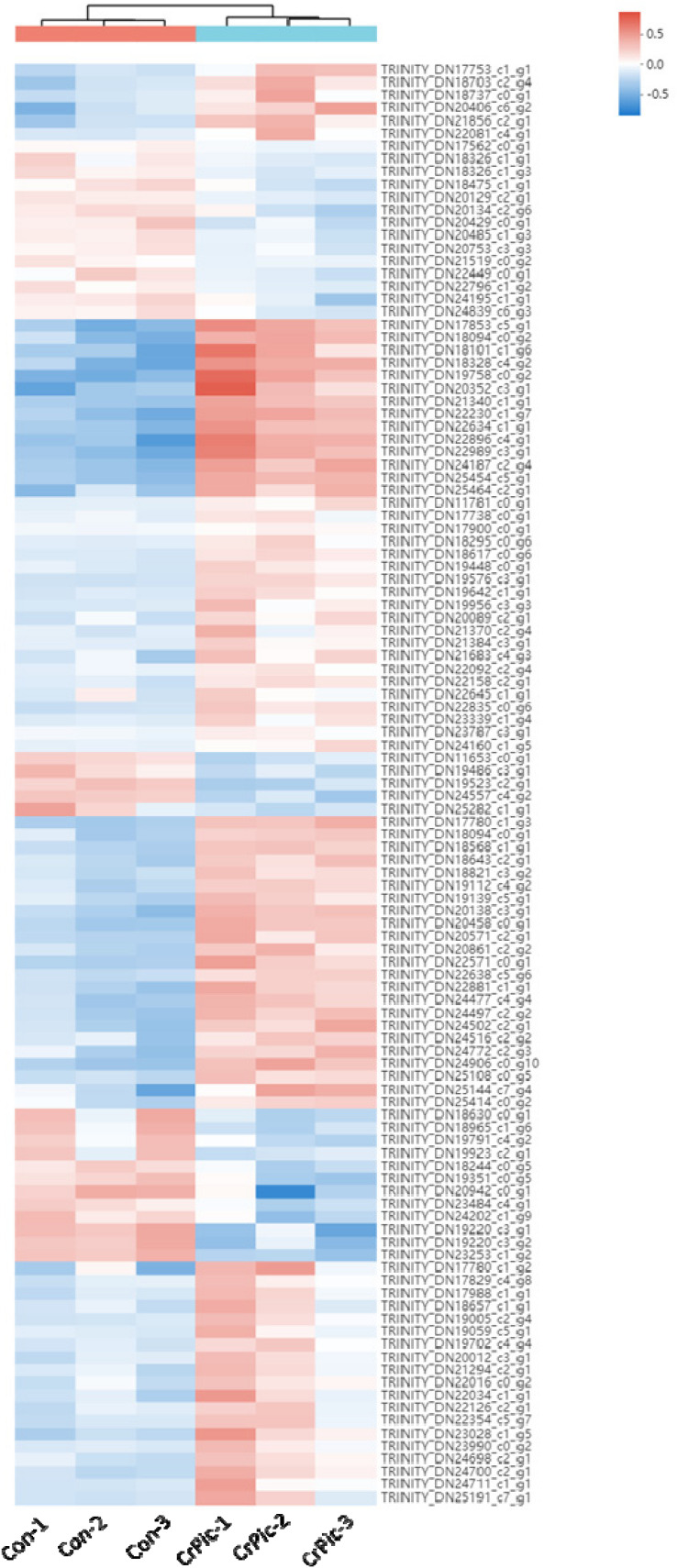
Heat map analysis of 113 differential expression genes (DEGs) in the livers of *Plectropomus leopardus* from Control and CrPic treatment. All of the genes were expressed differentially in between Control and CrPic group (selected by adjusted *p* < 0.05). The control groups were Con_1, Con_2, and Con_3. The treatment groups were CrPic_1, CrPic_2, and CrPic_3. The color bar from blue to red corresponds to gene expression levels increasing from low to high.

There were 113 differential expression genes [31 down-regulated and 82 up-regulated DEGs (Supplementary Figure 1)] obtained based on *p* < 0.05 and the gene expression values of >twofold change (Figure 1 and Supplementary Table 4).

### GO, COG, KEGG Functional Classification Analysis for DEGs

**FIGURE 2:**
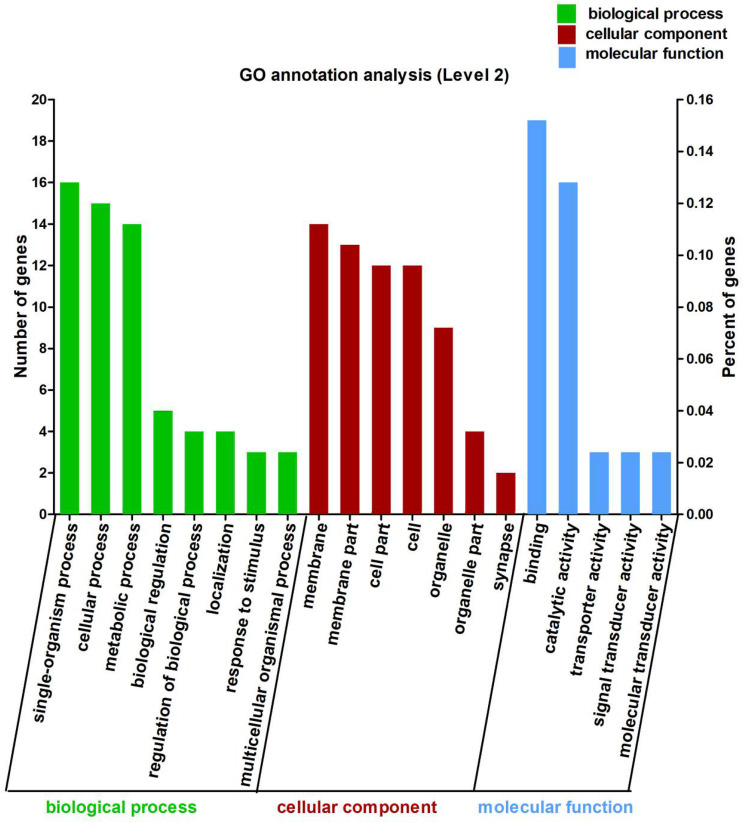
Gene Ontology (GO) annotation analysis of the 113 differentially expressed genes (DEGs) between Control and CrPic treatment. The top 20 GO terms were enriched as biological process (BP, green bars), cellular components (CC, red bars), or molecular function (MF, blue bars). The numbers of differentially expressed genes between Control and CrPic group in each category were compared. The horizontal axis represents three major functional categories of GO terms including BP, CC and MF, the left vertical axis shows the number of DEGs, and the right vertical axis shows the percent of DEGs.

In this study, the 113 DEGs annotated in GO were assigned to three categories, which were biological process (62.83%), cellular component (63.72%), and molecular function (38.94%) (Figure 2). These DEGs were further divided into 20 subcategories (Supplementary Table 5).

Using GO in the DAVID bioinformatics software (version 6.8) to analyze the down-regulated and up-regulated DEGs, we found that 12 DEGs were involved in cellular process (10.62%), whilst 14 DEGs each were found for single-organism process (12.39%), catalytic activity (12.39%), and binding (12.39%). Interestingly, we further found that these DEGs were all up-regulated and involved in the main abundant subcategories (Supplementary Figure 2).

**FIGURE 3:**
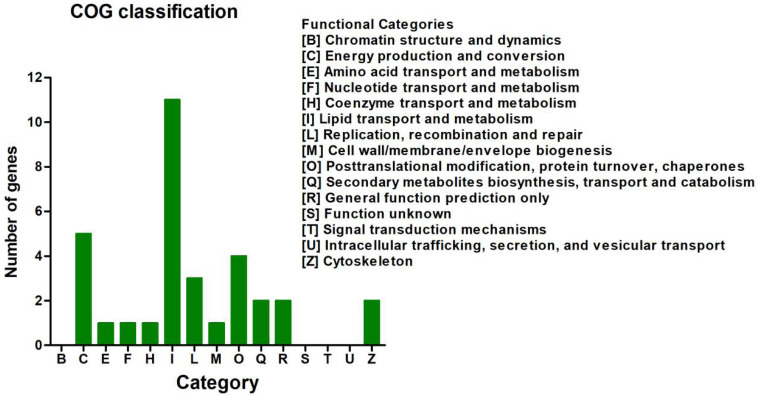
Clusters of Orthologous Groups of proteins (COG) classification analysis of the 113 differentially expressed genes (DEGs). The 113 DEGs were classified into 15 functional categories. The vertical axis shows the number of DEGs in a definite functional cluster. The horizontal axis shows the function class.

In the COG database, the 113 DEGs were classified into 15 functional categories (Figure 3 and Supplementary Table 6). The predominant category was the cluster for lipid transport and metabolism (9.73%), followed by energy production and conversion (4.42%) and post-translational modification, protein turnover, and chaperones (3.54%) (Supplementary Table 6).

**FIGURE 4:**
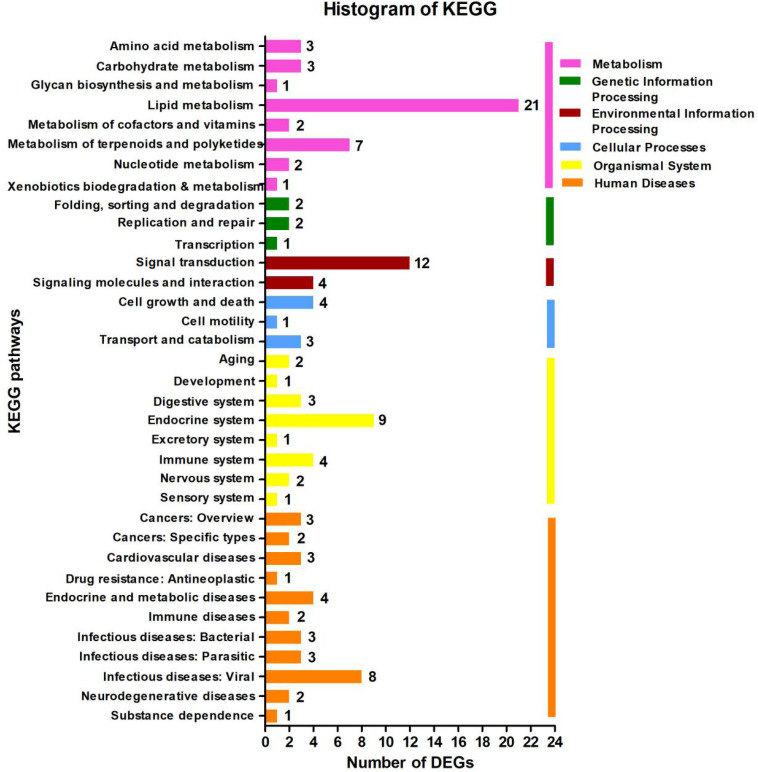
Kyoto Encyclopedia of Genes and Genomes (KEGG) pathway classification analysis of the liver’s transcripts in *Plectropomus leopardus*. The vertical axis shows the name of the KEGG metabolic pathway, and the horizontal axis shows the number of the differentially expressed genes (DEGs) annotated in the pathway. There are six categories in KEGG metabolic pathway, including Metabolism (pink), Genetic Information Processing (green), Environmental Information Processing (dark red), Cellular Processes (blue), Organismal Systems (yellow), and Human Diseases (orange).

In the KEGG database, the 113 DEGs were classified into six major categories with 35 subclasses (Figure 4 and Supplementary Table 7). The major categories were metabolism (35.4%), human diseases (28.32%), organismal systems (20.35%), environmental information processing (14.16%), cellular processes (7.08%), and genetic information processing (4.42%). Here, we found that the number of DEGs was robust for the lipid metabolism pathway (18.58%) (Figure 4), and signal transduction also featured strongly (10.62%), the affected pathways included calcium signaling [cholinergic receptor, muscarinic 5 (*chrm5*), mitochondrial calcium uniporter dominant negative subunit beta (*mcub*), purinergic receptor P2X, ligand gated ion channel 7 (*p2rx7*)], MAPK signaling [dual-specificity phosphatase 1 (*dusp1*), heat shock protein 70 (*hsp70*), interleukin-1 receptor type 1 (*IL-1R1*), transforming growth factor beta-3 (*tgfb3*)], FoxO signaling (phosphoenolpyruvate carboxykinase (*pepck*), *tgfb3*), AMPK signaling [acyl-CoA desaturase (*acod*), 3-hydroxy-3-methylglutaryl-CoA reductase (*hmgcr*), *pepck*], ErbB signaling [heparin-binding EGF-like growth factor (*hbegf*)], and Wnt signaling [chromodomain-helicase-DNA-binding protein 8 (*chd8*)]. Among those genes, *chrm5, tgfb3*, and *acod* were down-regulated, and *IL-1R1, mcub, p2rx7, dusp1, hsp70, pepck, hmgcr*, and *hbegf* were up-regulated (Figure 4 and Supplementary Table 4). We also observed that for lipid metabolism, 18 out of 21 DEGs were up-regulated (Supplementary Figure 3).

### KEGG and GO Enrichment Analysis of DEGs

**FIGURE 5:**
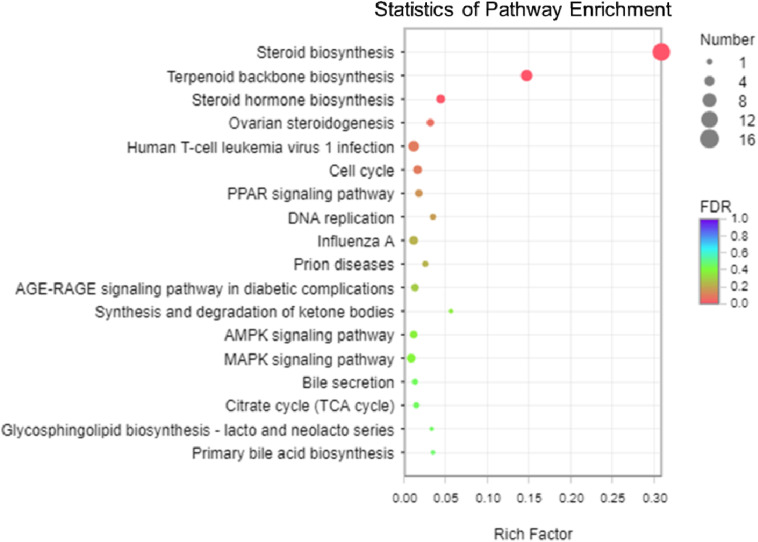
The scatter plot of Kyoto Encyclopedia of Genes and Genomes (KEGG) pathway enrichment analysis among the 113 differentially expressed genes (DEGs). The vertical axis shows the categories in the pathway, and the horizontal axis shows the enrichment factor. The point size shows the number of DEGs in the pathway. The point color shows different *Q*-values (FDR). Steroid biosynthesis contained the most DEGs.

The analysis of KEGG enrichment revealed that top of 18 pathways and three of these pathways were significantly enriched (*p* < 0.05, Fisher’s exact test followed by Bonferroni), namely those for steroid biosynthesis, terpenoid backbone biosynthesis, and steroid hormone biosynthesis (Figure 5 and Supplementary Figures 5, 6). To confirm these significant enrichment pathways, we conducted a GO enrichment analysis and observed six significantly enriched pathways (*p* < 0.05, Supplementary Figure 4), including those for lipid biosynthesis, isoprenoid biosynthesis, lipid metabolic processes, isoprenoid metabolic processes, single-organism biosynthetic processes, and cellular lipid metabolic processes. These results showed that lipid metabolism is involved in the significantly enriched pathways, which are strongly correlated with the KEGG functional classification analysis.

**FIGURE 6:**
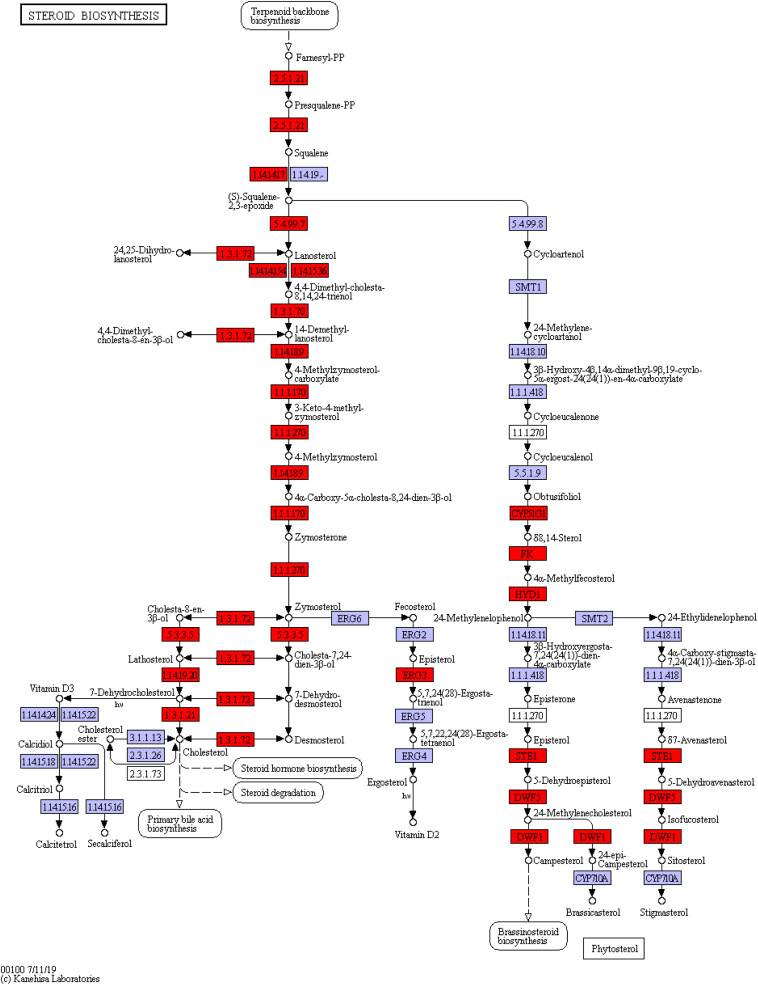
Steroid biosynthesis pathway enriched in the liver of *Plectropomus leopardus*. The red boxes represented the significantly up-regulated genes involved in the pathway. The expression levels of genes encoding 7-dehydrocholesterol reductase (*dhcr7*), delta14-sterol reductase (*tm7sf*), delta7-sterol 5-desaturase (*sc5dl*), squalene monooxygenase (*sqle*), farnesyl-diphosphate farnesyltransferase (*fdft1*), cholestenol Delta-isomerase (*ebp*), lanosterol synthase (lss), sterol 14alpha-demethylase (*cyp51*), sterol-4alpha-carboxylate 3-dehydrogenase (decarboxylating) (*nsdhl*), methylsterol monooxygenase (*me*s*ol*), delta24-sterol reductase (*dhcr24*), 17 beta-estradiol 17-dehydrogenase/3 beta-hydroxysteroid 3-dehydrogenase (*hsd17b7*), all of which were involved in steroid biosynthesis and increased significantly under CrPic treatment.

Among the three pathways showing significantly enriched DEGs, steroid biosynthesis (*p* = 2.85 × 10^–9^) was the most significant pathway (Supplementary Table 8). There were 12 genes involved in steroid biosynthesis that were significantly up-regulated under dietary CrPic treatment, including dehydrocholesterol reductase (*dhcr7*, *dhcr24*), emopamil binding protein (*ebp*), lanosterol synthase (*lss*), methylsterol monooxygenase 1 (*msmo1*), squalene epoxidase (*sqle*), and sterol 14-alpha demethylase (*cyp51*), delta14-sterol reductase (*tm7sf2*), delta7-sterol 5-desaturase (*sc5dl*), farnesyl-diphosphate farnesyltransferase (*fdft1*), sterol-4alpha-carboxylate 3-dehydrogenase (decarboxylating) (*nsdhl*), and hydroxysteroid 17-beta dehydrogenase 7 (*hsd17b7*) (Figure 6).

### Verification of RNA-Seq Data Using qRT-PCR

**FIGURE 7:**
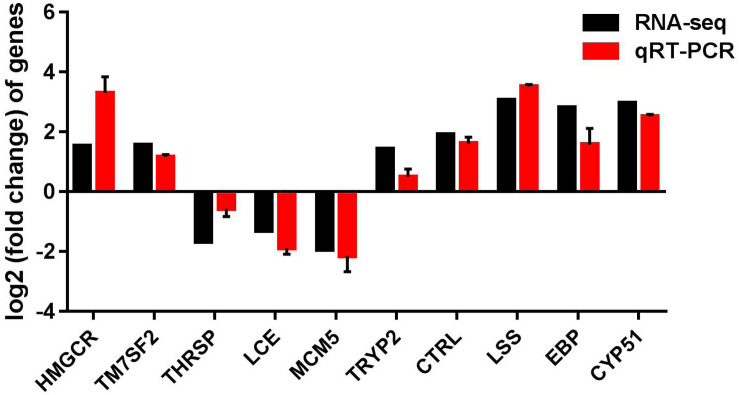
qRT-PCR validation of up-regulated and down-regulated genes analyzed by RNA-Seq. qRT-PCR was performed for 10 selected genes that were identified as differentially expressed between control and CrPic-treated group. The *Y*-axis shows the relative mRNA expression levels.

To validate the reliability of transcriptome sequencing data obtained from Illumina analysis in our study, we chose 10 differentially expressed genes (DEGs) for qRT-PCR analysis, using the same RNA samples. These DEGs include hydroxy-3-methylglutaryl-CoA reductase (HMGCR), delta14-sterol reductase (TM7SF2), thyroid hormone responsive (THRSP), low choriolytic enzyme (LCE), minichromosome maintenance complex component-5 (MCM5), trypsinogen-2 (TRYP2), and chymotrypsin like (CTRL), EBP, LSS, and CYP51. We found that the differential expression patterns of 10 selected genes are consistent with the RNA-seq results; gene expression of *hmgcr*, *ebp, lss, cyp51, tm7sf2, tryp2*, and *ctrl* were significantly increased, and gene expression of *thrsp, lce*, and *mcm5* were decreased (Figure 7), suggesting that the RNA-seq data are reliable.

## Discussion

Our study provided the first evidence that a long-term, high dose of dietary CrPic exposure significantly affects lipid metabolism in aquatic animals at the gene expression level. The three most enriched pathways were steroid biosynthesis, terpenoid backbone biosynthesis, and steroid hormone biosynthesis.

### Steroid Biosynthesis Was Up-Regulated by Dietary CrPic Exposure

Steroid biosynthesis is a well-known component of lipid metabolism in animals (Si et al., 2018), and it plays a vital role in responding to environmental pressure of aquatic animals (e.g., salinity change: Xu et al., 2015). We found evidence that the steroid biosynthesis was significantly affected by dietary CrPic supplementation in marine fish by identifying several steroid biosynthesis-related genes that were up-regulated by CrPic exposure (e.g., *ebp*, *lss*, *dhcr7*, *dhcr24*, *msmo1*, *sqle*, *cyp51*). As far as we know, this is the first study showing that dietary CrPic has an effect on the expression of steroid biosynthesis-related genes in fish.

The *ebp* gene, specifically, codes for the enzyme sterol isomerase, which plays an important function in regulating the pathway of sterol biosynthesis in animals (Sanchez-Pulido and Ponting, 2014). Sterols act as an important compounds, and it works in lots of biological processes, such as possessing transport capability and acting as cell membrane components (Schaller, 2003). In this study, we observed that the expression of the *ebp* gene was up-regulated in response to dietary CrPic exposure, suggesting an increased capacity to stimulate biosynthesis of sterols in the livers of *P. leopardus*. In addition, *lss* gene transcription was also increased under dietary CrPic exposure. The *lss* gene encodes the enzyme lanosterol synthase, which is a key enzyme in the cholesterol biosynthetic pathway. Lanosterol is one of the upstream precursors in the pathway of sterol biosynthesis for many animals (Chen et al., 2007). Increased *lss* expression thus indicated enhanced synthesis of cholesterol in the livers of *P. leopardus*.

The *cyp51* gene specifically encodes for the lanosterol 14-alpha demethylase protein, which is a key enzyme for the conversion of lanosterol to cholesterol, and thus acts as an important component in lipid metabolism and the steroid biosynthesis pathway (Lewinska et al., 2014). Long-term exposure of dietary CrPic led to the up regulation of *cyp51*, indicating active cholesterol synthesis in *P. leopardus*. Conversely, other studies have reported that long-term exposure of difenoconazole down-regulates the expression of sterol-genesis genes (e.g., *cyp51*) in zebrafish (Mu et al., 2015). While another study reported that the *lss* and *dhcr24* (24-dehydrocholesterol reductase) gene expressions were up-regulated in the steroid biosynthesis pathway in Nile tilapia (*O. niloticus*) under salinity stress (Xu et al., 2015). Long-term exposure to dietary CrPic up regulated both of these genes in *P. leopardus*, lending further evidence that liver cells were required to increase cholesterol synthesis in response to long-term Cr exposure and that this was likely a stress response. An interesting and recent study found that eight genes (FDFT1, SQLEA, LSS, MSMO1, NSDHL, EBP, SC5D, DHCR7) were remarkably up-regulated in the steroid biosynthesis pathway under low temperature (12 and 14°C) (Shi et al., 2020). These results strongly indicate that up-regulation of the steroid biosynthesis related genes may play an important role in Cr defense and different tolerance responses (e.g., cold stress, salinity stress).

### Terpenoid Backbone Biosynthesis Was Up-Regulated by Dietary CrPic Exposure

The terpenoid backbone biosynthesis pathway was first found to be up-regulated by dietary CrPic intake in our study. Terpenoids (e.g., sterols and steroids) are the most abundant precursors and lipids in complex secondary metabolite synthesis (Tholl, 2015). Terpenoids production needs three key enzymes [i.e., phosphomevalonate kinase (*pmvk*), 3-hydroxy-3-methylglutaryl-CoA synthase 1 (*hmgcs1/hmgcr*), and farnesyl diphosphate synthease (*fdps*)], the *hmgcs1/hmgcr* and *pmvk* genes code for well-known enzymes, those enzymes involved in biosynthesis of mevalonate pathway, which is potentially the only pathway to produce dimethylallyl-PP and isopentenyl-PP precursors and plays a critical role in terpenoid backbone biosynthesis (Si et al., 2018). Subsequently, dimethylallyl diphosphate (DMAPP) and isopentenyl pyrophosphate (IPP) transformed to farnesyl-PP (requiring the product of *fpp*—farnesyl pyrophosphate synthase), and farnesyl diphosphate synthease helps produce terpenoids (the product of *fdps*; Wasko et al., 2011). Previous studies have shown that the biosynthesis pathway of the terpenoid backbone and steroid were activated in the brain of grass carp (*Ctenopharyngodon idellus*) under low temperatures conditions. They found that six genes (HMGCR,HMGCS1, ACAT2, MVD, IDI1, MVK) were up-regulated at 12 and 14°C in the terpenoid backbone biosynthesis pathway (Shi et al., 2020). Consistent with previous studies, we found that five genes (HMGCR, PMVK, MVD, IDI, FDPS) were significantly up-regulated in the terpenoid backbone biosynthesis pathway under organic Cr (III) [as chromium picolinate (CrPic)] exposure; some of the selected genes (HMGCR, EBP, LSS) were verified by qRT-PCR. Another study also showed that the expression of terpenoid backbone and steroid biosynthesis were affected in the liver of Atlantic salmon (*Salmo salar*) by heat stress. They found that *hmgcr* was down-regulated after 24 h of heat stress in terpenoid backbone biosynthesis, and they also found that the expression of genes (*fdft, lss sc5d, sqle*) associated with the steroid biosynthesis pathway were reduced (Shi et al., 2019). In the present study, we found the related gene expressions of those key enzymes involved in the terpenoid backbone biosynthesis pathway and steroid biosynthesis pathway, were all up-regulated by CrPic exposure. Therefore, the up-regulation of these enzymes, related to terpenoids biosynthesis and steroid biosynthesis, showed a requirement for increased lipid metabolism in the fish exposed to dietary CrPic.

### Steroid Hormone Biosynthesis Was Up-Regulated by Dietary CrPic Exposure

Steroid hormones play a critical role in regulating stress responses, water and salt balance, and nutrient metabolism (Schiffer et al., 2019). Hydroxysteroid dehydrogenase (*hsd*) and cytochrome P450 (*cyp*) enzymes are two major enzymes involved in steroid hormone biosynthesis (Schiffer et al., 2019). Hydroxysteroid 17-beta dehydrogenase 7 (*hsd17b7*) and cytochrome P450 family 7 subfamily A member 1 (*cyp7a1*), are important enzymes required for steroid hormone biosynthesis. In our study, we found that the expression of *cyp7a1* and *hsd17b1* genes were up-regulated. These enzymes are vital to the biosynthesis of steroid hormones, indicating that the steroid hormone biosynthesis of the fish was significantly stimulated by dietary CrPic supplementation. This is the first report to indicate that steroid hormone biosynthesis is influenced under dietary CrPic exposure in teleost fish. Both *cyp17a1* and *hsd3b2* are key components of cortisol production, a hormone produced in response to various pressure backgrounds, it can be used as a biomarker when aquatic animals respond to stress (Tokarz et al., 2015). A previous study has confirmed that fish homeostasis under cutaneous stress increased cortisol levels (Kulczykowska et al., 2018), suggesting that long-term Cr exposure causes stress to *P. leopardus.*

### Signal Transduction Was Indispensable in Lipid Metabolism Disorder

In the present study, in the CrPic group, genes from several cellular regulatory pathways related to signal transduction were expressed differentially. The affected pathways included calcium signaling (*chrm5, mcub, p2rx7*), MAPK signaling (*dusp1, hsp70, IL-1R1, tgfb3*), FoxO signaling (*pepck, tgfb3*), AMPK signaling (*acod, hmgcr, pepck*), ErbB signaling (*hbegf*), and Wnt signaling (*chd8*).

Mitochondria performs critical roles in many cell signaling pathways. Several mechanisms are involved, including altered Ca^2+^ levels and reactive oxygen species (ROS) generation (Chandel, 2014). In our results, the expression of a gene central to the regulation of Ca^2+^ homeostasis in mitochondria and the cytosol, the *mcub* gene, was up regulated. The product of this gene negatively regulates the activity of the mitochondrial Ca^2+^ uniporter (MCU), decreasing Ca^2+^ transport into the mitochondria (Mammucari et al., 2018). This can increase cytosolic Ca^2+^, which activates signaling pathways that regulate many cellular processes (Bohovych and Khalimonchuk, 2016). At the same time, Ca^2+^ in the mitochondrial matrix is decreased, limiting the production of ROS (Jhun et al., 2016) due to the decreased activity of key Ca dependent enzymes that are required for oxidative metabolism in the mitochondria (Mammucari et al., 2018). The main production of ROS in the cell is mitochondria, and reduced ROS production by this organelle will affect the many cellular processes regulated through ROS (Chandel, 2014). Moreover, in mammal cardiomyocytes, *MCUb* was up-regulated after injury, apparently as a stress response (Lambert et al., 2019), which was consistent with the transcription level response observed in the fish liver cells in response to stress caused by dietary CrPic intake in this study.

Overall, the changes in transcription level expression of the genes observed in our study suggests that the cells of the fish liver adapted to dietary CrPic exposure by altering several signaling pathways, which could in turn potentially affect many aspects of the liver’s cell functions and biochemistry.

## Conclusion

In conclusion, the present study successfully constructed a database of transcriptome changes in juvenile coral trout under long-term, high dose exposure to dietary CrPic. We identified 113 differentially expressed genes (DEGs) significantly enriched in the CrPic treatment. Our study demonstrates that the RNA-Seq analysis is highly effective in determining molecular/cellular level responses to potentially toxic contaminants in aquatic animals. The findings here demonstrate that dietary CrPic exposure significantly up-regulates lipid metabolism in teleost fish at the transcription level, mainly by affecting steroid and terpenoid backbone biosynthesis pathways, and altered signal transduction pathways. This suggests that a high dose of organic dietary Cr(III) exposure results in stress and sub-lethal toxic effects in aquatic animals. Cautious health risk assessment of dietary Cr(III) intake is therefore highly recommended in both commercial and natural diets for aquatic animals, which has previously been largely ignored.

## Data Availability Statement

The datasets presented in this study can be found in online repositories. The names of the repository/repositories and accession number(s) can be found below: https://www.ncbi.nlm.nih.gov/, PRJNA684012.

## Ethics Statement

The animal study was reviewed and approved by the Hainan University Institutional Animal Use and Care Committee (Hainan, China).

## Author Contributions

LW analyzed all the data, wrote, and revised the manuscript. YL and HY performed the qRT-PCR experiment and collected the materials. JX, CH, and IG revised the manuscript. ZG and DH designed the study and revised the manuscript. All authors have read and approved the final manuscript.

## Conflict of Interest

The authors declare that the research was conducted in the absence of any commercial or financial relationships that could be construed as a potential conflict of interest.

## Footnotes

1 https://github.com/trinityrnaseq/trinityrnaseq

2 http://web.expasy.org/docs/swiss-prot_guideline.html

3 ftp://ftp.ncbi.nlm.nih.gov/blast/db/

4 http://www.ncbi.nlm.nih.gov/COG/

5 http://pfam.xfam.org/

6 http://www.genome.jp/kegg/

7 http://www.geneontology.org/

## References

[B1] Ahmed A. R. Jha A. N. Davies S. J. (2012). The efficacy of chromium as a growth enhancer for mirror carp (*Cyprinus carpio* L): an integrated study using biochemical, genetic, and histological responses. *Biol. Trace. Elem. Res.* 148 187–197. 10.1007/s12011-012-9354-4 22351105

[B2] Andersson M. Grawe K. P. Karlsson O. Abramssonzetterberg L. Hellman B. (2007). Evaluation of the potential genotoxicity of chromium picolinate in mammalian cells in vivo and in vitro. *Food. Chem. Toxicol.* 45 1097–1106. 10.1016/j.fct.2006.11.008 17418471

[B3] Bai Y. Zhao X. Qi C. Wang L. Cheng Z. Liu M. (2014). Effects of chromium picolinate on the viability of chick embryo fibroblast. *Hum. Exp. Toxicol.* 33 403–413. 10.1177/0960327113499042 23925942

[B4] Bohovych I. Khalimonchuk O. (2016). Sending out an SOS: mitochondria as a signaling hub. *Front Cell Dev Biol.* 4:109. 10.3389/fcell.2016.00109 27790613 PMC5061732

[B5] Chandel N. S. (2014). Mitochondria as signaling organelles. *BMC Biol.* 12:34. 10.1186/1741-7007-12-34 24884669 PMC4035690

[B6] Chen L. L. Wang G. Z. Zhang H. Y. (2007). Sterol biosynthesis and prokaryotes-to-eukaryotes evolution. *Biochem. Biophys. Res. Commun.* 363 885–888. 10.1016/j.bbrc.2007.09.093 17923113

[B7] Fan W. Liu A. Wang W. Zheng G. Teng A. (2012). Hepatoprotective activity of CrPic against alloxan-induced hepatotoxicity in mice. *Biol. Trace. Elem. Res.* 149 227–233. 10.1007/s12011-012-9415-8 22528782

[B8] FDA (2005). *FDA US Qualified Health Claim Finds CrPic Safe for Intended Use. 14639-25-9 (CAS DataBase Reference).* Available online at: https://www.fda.gov/food/news-events-cfsan/cfsan-constituent- updates (accessed Septemper 12, 2019).

[B9] Guo Z. Ni Z. Ye H. Xiao J. Chen L. Green I. (2019). Simultaneous uptake of Cd from sediment, water and diet in a demersal marine goby Mugilogobius chulae. *J Hazard Mater.* 364 143–150. 10.1016/j.jhazmat.2018.09.045 30343176

[B10] Hamilton E. M. Young S. D. Bailey E. H. Watts M. J. (2018). Chromium speciation in foodstuffs: a review. *Food. Chem.* 250 105–112. 10.1016/j.foodchem.2018.01.016 29412899

[B11] Hepburn D. D. Xiao J. Bindom S. Vincent J. B. Odonnell J. M. (2003). Nutritional supplement chromium picolinate causes sterility and lethal mutations in *Drosophila melanogaster*. *Proc. Natl. Acad. Sci. U.S.A.* 100 3766–3771. 10.1073/pnas.0636646100 12649323 PMC152996

[B12] IUCN (2004). *Red list of Threatened Species: Plectropomus Leopardus*. e.T44684A10924940. Available online at: www.iucnredlist.org. (accessed February 19, 2020).

[B13] Jhun B. S. Mishra J. Monaco S. Fu D. Jiang W. Sheu S. (2016). The mitochondrial Ca^2+^ uniporter: regulation by auxiliary subunits and signal transduction pathways. *Am. J. Physiol. Cell. Physiol.* 311 C67–C80. 10.1152/ajpcell.00319.2015 27122161 PMC4967134

[B14] Jiang L. Vincent J. B. Bailey M. M. (2018). [Cr_3_O(O_2_CCH_2_CH_3_)_6_(H_2_O)_3_]NO_3_⋅H_2_O (Cr3) Toxicity potential in bacterial and mammalian cells. *Biol. Trace. Elem. Res.* 183 342–350. 10.1007/s12011-017-1132-x 28879636

[B15] Kim J. H. Kang J. C. (2016). Oxidative stress, neurotoxicity, and metallothionein (MT) gene expression in juvenile rock fish *Sebastes schlegelii* under the different levels of dietary chromium (Cr(6+)) exposure. *Ecotoxicol. Environ. Saf.* 125 78–84. 10.1016/j.ecoenv.2015.12.001 26680530

[B16] Krol E. Krejpcio Z. Okulicz M. Smigielska H. (2020). Chromium(III) glycinate complex supplementation improves the blood glucose level and attenuates the tissular copper to zinc ratio in rats with mild hyperglycaemia. *Biol. Trace. Elem. Res.* 193 185–194. 10.1007/s12011-019-01686-7 30826908 PMC6914712

[B17] Kulczykowska E. Kalamarzkubiak H. Gozdowska M. Sokolowska E. (2018). Cortisol and melatonin in the cutaneous stress response system of fish. *Comp. Biochem. Physiol. A.* 218 1–7. 10.1016/j.cbpa.2018.01.003 29355753

[B18] Lambert J. P. Luongo T. S. Tomar D. Jadiya P. Gao E. Zhang X. (2019). MCUB Regulates the molecular composition of the mitochondrial calcium uniporter channel to limit mitochondrial calcium overload during stress. *Circulation* 140 1720–1733. 10.1161/CIRCULATIONAHA.118.037968 31533452 PMC6996560

[B19] Lewinska M. Juvan P. Perse M. Jeruc J. Kos S. Lorbek G. (2014). Hidden disease susceptibility and sexual dimorphism in the heterozygous knockout of Cyp51 from cholesterol synthesis. *PLos One* 9:e112787. 10.1371/journal.pone.0112787 25393872 PMC4231084

[B20] Li H. Meng X. Wan W. Liu H. Sun M. Wang H. (2018). Effects of chromium picolinate supplementation on growth, body composition, and biochemical parameters in Nile tilapia *Oreochromis niloticus*. *Fish. Physiol. Biochem.* 44 1265–1274. 10.1007/s10695-018-0514-0 29961187

[B21] Mammucari C. Raffaello A. Vecellio Reane D. Gherardi G. De Mario A. Rizzuto R. (2018). Mitochondrial calcium uptake in organ physiology: from molecular mechanism to animal models. *Pflugers Arch.* 470 1165–1179. 10.1007/s00424-018-2123-2 29541860 PMC6060757

[B22] Marmett B. Nunes R. B. De Souza K. S. Dal Lago P. Rhoden C. R. (2018). Aerobic training reduces oxidative stress in skeletal muscle of rats exposed to air pollution and supplemented with chromium picolinate. *Redox. Rep.* 23 146–152. 10.1080/13510002.2018.1475993 29776315 PMC6748694

[B23] Mayorga E. J. Kvidera S. K. Seibert J. T. Horst E. A. Abuajamieh M. Al-Qaisi M. (2018). Effects of dietary chromium propionate on growth performance, metabolism, and immune biomarkers in heat-stressed finishing pigs. *J. Anim. Sci.* 97 1185–1197. 10.1093/jas/sky484 30590717 PMC6396268

[B24] Mu X. Y. Wang K. Chai T. T. Zhu L. Z. Yang Y. Zhang J. (2015). Sex specific response in cholesterol level in zebrafish (*Danio rerio*) after long-term exposure of difenoconazole. *Environ. Pollut.* 197 278–286. 10.1016/j.envpol.2014.11.019 25483594

[B25] NIH (2013). *National Institute of Health: Office of Dietary Supplements. Chromium: Dietary Supplement Fact Sheet.* Available online at: https://ods.od.nih.gov/index.aspx (accessed March 18, 2020).

[B26] Peng Z. Qiao W. Wang Z. Dai Q. He J. Guo C. (2010). Chromium improves protein deposition through regulating the mRNA levels of IGF-1, IGF-1R, and Ub in rat skeletal muscle cell. *Biol. Trace. Elem. Res.* 137 226–234. 10.1007/s12011-009-8579-3 20013160

[B27] Pfaffl M. W. (2001). A new mathematical model for relative quantification in real-time RT-PCR. *Nucleic. Acids. Res.* 29 45–51. 10.1093/nar/29.9.e45 11328886 PMC55695

[B28] Pires K. A. Santos D. C. Graca D. S. Melo M. M. Barbosa F. A. Sotoblanco B. (2015). Effects of two sources of chromium on performance, blood and liver lipid levels in Nile Tilapia (*Oreochromis niloticus*). *Acta. Sci. Vet.* 43 1–8.

[B29] Qiao W. Peng Z. Wang Z. Wei J. Zhou A. (2009). Chromium improves glucose uptake and metabolism through upregulating the mRNA levels of IR, GLUT4, GS, and UCP3 in skeletal muscle cells. *Biol. Trace. Elem. Res.* 131 133–142. 10.1007/s12011-009-8357-2 19283340

[B30] Refaie F. M. Esmat A. Y. Mohamed A. F. Nour W. H. (2009). Effect of chromium supplementation on the diabetes induced-oxidative stress in liver and brain of adult rats. *Biometals.* 22 1075–1087. 10.1007/s10534-009-9258-8 19693677

[B31] Ren M. Mokrani A. Liang H. Ji K. Xie J. Ge X. (2018). Dietary chromium picolinate supplementation affects growth, whole-body composition, and gene expression related to glucose metabolism and lipogenesis in juvenile Blunt Snout Bream, *Megalobrama amblycephala*. *Biol. Trace. Elem. Res.* 185 205–215. 10.1007/s12011-018-1242-0 29344818

[B32] Sanchez-Pulido L. Ponting C. P. (2014). TM6SF2 and MAC30, new enzyme homologs in sterol metabolism and common metabolic disease. *Front. Genet.* 5:439. 10.3389/fgene.2014.00439 25566323 PMC4263179

[B33] Schaller H. (2003). The role of sterols in plant growth and development. *Prog. Lipid. Res.* 42 163–175. 10.1016/s0163-7827(02)00047-4 12689617

[B34] Schiffer L. Barnard L. Baranowski E. S. Gilligan L. C. Taylor A. E. Arlt W. (2019). Human steroid biosynthesis, metabolism and excretion are differentially reflected by serum and urine steroid metabolomes: a comprehensive review. *J. Steroid. Biochem. Mol. Biol.* 194 105439. 10.1016/j.jsbmb.2019.105439 31362062 PMC6857441

[B35] Selcuk Z. Tiril S. U. Alagil F. Belen V. Salman M. Cenesiz S. (2010). Effects of dietary l-carnitine and chromium picolinate supplementations on performance and some serum parameters in rainbow trout (*Oncorhynchus mykiss*). *Aquacult. Int.* 18 213–221. 10.1007/s10499-008-9237-z

[B36] Shafik N. M. Baalash A. Ebeid A. M. (2017). Synergistic cardioprotective effects of combined chromium picolinate and atorvastatin treatment in triton X-100-induced hyperlipidemia in rats: impact on some biochemical markers. *Biol. Trace. Elem. Res.* 180 255–264. 10.1007/s12011-017-1010-6 28409410

[B37] Shi K. Dong S. Zhou Y. Li Y. Gao Q. Sun D. (2019). RNA-seq reveals temporal differences in the transcriptome response to acute heat stress in the Atlantic salmon (*Salmo salar*). *Comp. Biochem. Physiol. Part D Genom Proteom.* 30 169–178. 10.1016/j.cbd.2018.12.011 30861459

[B38] Shi M. J. Zhang Q. X. Li Y. M. Zhang W. T. Liao L. J. Cheng Y. Y. (2020). Global gene expression profile under low temperature conditions in the brain of the grass carp (*Ctenopharyngodon idellus*). *PLoS One* 15:e0239730. 10.1371/journal.pone.0239730 32976524 PMC7518592

[B39] Si Y. Wen H. Li Y. He F. Li J. Li S. (2018). Liver transcriptome analysis reveals extensive transcriptional plasticity during acclimation to low salinity in *Cynoglossus semilaevis*. *BMC. Genomics.* 19:464. 10.1186/s12864-018-4825-4 29914359 PMC6006554

[B40] Staniek H. Wojcik R. W. (2018). The combined effects of iron excess in the diet and chromium(III) supplementation on the iron and chromium status in female rats. *Biol. Trace. Elem. Res.* 184 398–408. 10.1007/s12011-017-1203-z 29164513 PMC6061187

[B41] Stoecker B. (1999). Chromium absorption, safety, and toxicity. *J. Trace. Elem. Exp. Med.* 12 163–169. 10.1002/(SICI)1520-670X199912:2<163::AID-JTRA13<3.0.CO;2-3 29214320

[B42] Tan G. Zheng S. Zhang M. Feng J. Xie P. Bi J. (2008). Study of oxidative damage in growing-finishing pigs with continuous excess dietary chromium picolinate intake. *Biol. Trace. Elem. Res.* 126 129–140. 10.1007/s12011-008-8207-7 18704273

[B43] Tholl D. (2015). Biosynthesis and biological functions of terpenoids in plants. *Adv. Biochem. Eng. Biotechnol.* 148 63–106. 10.1007/10_2014_295 25583224

[B44] Tian H. Guo X. Wang X. He Z. Sun R. Ge S. (2013). Chromium picolinate supplementation for overweight or obese adults. *Cochrane Database Syst. Rev.* 11:CD010063. 10.1002/14651858.CD010063 PMC7433292 24293292

[B45] Tokarz J. Moller G. Hrabe, de, Angelis M. Adamski J. (2015). Steroids in teleost fishes : a functional point of view. *Steroids.* 103 123–144. 10.1016/j.steroids.2015.06.011 26102270

[B46] Vincent J. B. (2000). Elucidating a biological role for chromium at a molecular level. *Acc. Chem. Res.* 33 503–510. 10.1021/ar990073r 10913239

[B47] Wang Y. Dong Y. Yao M. (2009). Chromium picolinate inhibits resistin secretion in insulin-resistant 3T3-L1 adipocytes via activation of AMP-activated protein kinase. *Clin. Exp. Pharmacol. Physiol.* 36 843–849. 10.1111/j.1440-1681.2009.05164.x 19298540

[B48] Wasko B. M. Smits J. P. Shull L. W. Wiemer D. F. Hohl R. J. (2011). A novel bisphosphonate inhibitor of squalene synthase combined with a statin or a nitrogenous bisphosphonate in vitro. *J. Lipid. Res.* 52 1957–1964. 10.1194/jlr.M016089 21903868 PMC3196227

[B49] Xu Z. Gan L. Li T. Xu C. Chen K. Wang X., et al. (2015). Transcriptome profiling and molecular pathway analysis of genes in association with salinity adaptation in Nile Tilapia *Oreochromis niloticus*. *PLoS One* 10:e0136506. 10.1371/journal.pone.0136506 26305564 PMC4548949

