# Supplementary material for: Dietary Trivalent Chromium Exposure Up-Regulates Lipid Metabolism in Coral Trout: The Evidence From Transcriptome Analysis
Source: Front Physiol. 2021 Feb 25;12:640898. doi: 10.3389/fphys.2021.640898 (PMC7959734; doi:10.3389/fphys.2021.640898)
Supplement: Supplementary file 1 [file Data_Sheet_1.pdf]

# Supplementary Materials

## Contents of Supporting Information

Table for the proximate composition of diets fed to the juvenile coral trout *Plectropomus leopardus*…………..…........................................................... Table S1

Table for the primers used for qRT-PCR……………………………………..Table S2

Table for the amplification efficiency of the tested targeted genes……..……..Table S3

Table for the list of 113 differential expression genes (DEGs) from *Plectropomus leopardus* in response to dietary CrPic exposure…………………………..Table S4

Table for the summary of Gene Ontology (GO) annotations of 113 differential expression genes (DEGs)………………………………………………......Table S5

Table for the summary of Clusters of Orthologous Groups of proteins (COG) classification analysis of 113 differential expression genes (DEGs)………Table S6

Table for the summary of Kyoto Encyclopedia of Genes and Genomes (KEGG) pathways analysis of 113 differential expression genes (DEGs)…………Table S7

Table for the significantly up/down-regulated pathways with their proteins Table S8

Figure for the volcano plot showed the gene expression differences between the Control and CrPic group……………………………………………..……Fig. S1

Figure for the heat map analysis of differentially expressed genes (DEGs) using Gene Ontology (GO) terms annotation cluster........................................................Fig. S2

Figure for the heat map analysis of 113 differential expression genes (DEGs) using Kyoto Encyclopedia of Genes and Genomes (KEGG) pathway…................. Fig. S3

Figure for the scatter plot showing Gene ontology (GO) enrichment among 113 differential expression genes (DEGs)…………………….………………….Fig. S4

Figure for the terpenoid backbone biosynthesis pathway……………………….Fig. S5

Figure for the steroid hormone biosynthesis pathway…………………….…….Fig. S6

**Table S1** The proximate composition of diets (mg kg$^{-1}$) fed to the juvenile coral trout (*Plectropomus leopardus)*. The control group diet was a commercial diet without extra Cr addition. The treatment group diet was commercial diet with supplementation of 200 mg kg$^{-1}$ Cr as chromium picolinate (CrPic). Values were mean ± SD (n=3).

| Proximate composition (in dry matter ) | control group diet | CrPic group diet |
|---|---|---|
| Cr concentration | 2.21 ± 0.14 | 195.67 ± 9.97 |
| Crude protein | 452.34 ± 33.52 | 440.16 ± 42.84 |
| Crude lipid | 104.46 ± 8.92 | 99.41 ± 10.95 |
| Crude ash | 128.64 ± 16.52 | 135.52 ± 20.81 |

**Table S2**. Primers used for qRT-PCR.

| gene | Forward (5'--3') | Reverse (5'--3') |
|---|---|---|
| *β-actin* | TACGAGCTGCCTGACGGACA | GGCTGTGATCTCCTTCTGCA |
| HMGCR | GGGGCTCGTTTCCTGAGTGA | CCTTGGAGTAGTCGTAGTCGGTGT |
| TM7SF2 | GAGACCATCGCCACAGCAAC | AGAAGTAAGGCAGGAGATGTGAGAA |
| THRSP | ACAGCACAGGAACAGGCAACC | AGGCTCGGCAGGAGAATGG |
| LCE | GGAACCTTGGCACTCCCTAT | ACTCATCTCCCTGGCCCTAC |
| MCM5 | CGCAGAGTCGCTGGCGAAGA | ACCTGGGAGCCGATAGCAAAA |
| TRYP2 | ACGAGGGCACAGAGCAGTTCA | CATCCAGGCACCTCAGACGAT |
| CTRL | TCGAAGCCATCGTGTTATCCT | GTTGTTGAAGTTCTGGGTGTTGTAG |
| LSS | CCTTTTCTCTCAGTGTGGTGC | ACCCTGTGGTGTCACTGTCG |
| EBP | TTCTACACCGAGCACAGGG | GCATCCACAATAAGCGTCA |
| CYP51 | ATGAGGATGGCTCGCTCCC | ACGGCACGTAGGCAAACTTCT |

**Table S3**. The amplification efficiency of the tested targeted genes.

| gene | Efficiency (E) |
|---|---|
| *β-actin* | 96.84% |
| HMGCR | 94.61% |
| TM7SF2 | 96.8% |
| THRSP | 97.82% |
| LCE | 94.85% |
| MCM5 | 98.92% |
| TRYP2 | 92.24% |
| CTRL | 96.58% |
| LSS | 97.72% |
| EBP | 92.86% |
| CYP51 | 95.96% |

**Table S4** The list of 113 differential expression genes (DEGs) from *Plectropomus leopardus* liver in response to dietary CrPic exposure (.xlsx 43kb)

**Table S5** Summary of Gene Ontology (GO) annotations of 113 differential expression genes (DEGs).

| Term Type | Term description | GO ID | Number of DEGs in category |
|---|---|---|---|
| Biological_process | cellular process | GO:0009987 | 15 |
| Biological_process | metabolic process | GO:0008152 | 14 |
| Biological_process | regulation of biological process | GO:0050789 | 4 |
| Biological_process | multicellular organismal process | GO:0032501 | 3 |
| Biological_process | multi-organism process | GO:0051704 | 1 |
| Biological_process | developmental process | GO:0032502 | 2 |
| Biological_process | biological regulation | GO:0065007 | 5 |
| Biological_process | localization | GO:0051179 | 4 |
| Biological_process | signaling | GO:0023052 | 1 |
| Biological_process | cellular component organization or biogenesis | GO:0071840 | 1 |
| Biological_process | response to stimulus | GO:0050896 | 3 |
| Biological_process | immune system process | GO:0002376 | 1 |
| Biological_process | locomotion | GO:0040011 | 1 |
| Biological_process | single-organism process | GO:0044699 | 16 |
| Cellular_component | synapse part | GO:0044456 | 2 |
| Cellular_component | cell junction | GO:0030054 | 1 |
| Cellular_component | cell part | GO:0044464 | 12 |
| Cellular_component | cell | GO:0005623 | 12 |
| Cellular_component | macromolecular complex | GO:0032991 | 2 |
| Cellular_component | organelle part | GO:0044422 | 4 |
| Cellular_component | membrane | GO:0016020 | 14 |
| Cellular_component | membrane part | GO:0044425 | 13 |
| Cellular_component | synapse | GO:0045202 | 2 |
| Cellular_component | organelle | GO:0043226 | 9 |
| Cellular_component | membrane-enclosed lumen | GO:0031974 | 1 |
| Molecular_function | transporter activity | GO:0005215 | 3 |
| Molecular_function | catalytic activity | GO:0003824 | 16 |
| Molecular_function | binding | GO:0005488 | 19 |
| Molecular_function | molecular transducer activity | GO:0060089 | 3 |
| Molecular_function | signal transducer activity | GO:0004871 | 3 |

**Table S6** Summary of Clusters of orthologous groups of proteins (COG) classification analysis of 113 differential expression genes (DEGs).

| Type | Functional Categories | 113DEGs_COG |
| --- | --- | --- |
| Information storage and processing | [B] Chromatin structure and dynamics | 0 |
| Metabolism | [C] Energy production and conversion | 5 |
| Metabolism | [E] Amino acid transport and metabolism | 1 |
| Metabolism | [F] Nucleotide transport and metabolism | 1 |
| Metabolism | [H] Coenzyme transport and metabolism | 1 |
| Metabolism | [I] Lipid transport and metabolism | 11 |
| Information storage and processing | [L] Replication, recombination and repair | 3 |
| Cellular processes and signaling | [M] Cell wall/membrane/envelope biogenesis | 1 |
| Cellular processes and signaling | [O] Posttranslational modification, protein turnover, chaperones | 4 |
| Metabolism | [Q] Secondary metabolites biosynthesis, transport and catabolism | 2 |
| Poorly characterized | [R] General function prediction only | 2 |
| Poorly characterized | [S] Function unknown | 0 |
| Cellular processes and signaling | [T] Signal transduction mechanisms | 0 |
| Cellular processes and signaling | [U] IntraCellular trafficking, secretion, and vesicular transport | 0 |
| Cellular processes and signaling | [Z] Cytoskeleton | 2 |

**Table S7** Summary of Kyoto Encyclopedia of Genes and Genomes (KEGG) pathways analysis of 113 differential expression genes (DEGs).

| First Category | Second Category | pathway ID | Description | Number of DEGs in category |
|---|---|---|---|---|
| Metabolism | Amino acid metabolism | map00310 | Lysine degradation | 1 |
| Metabolism | Amino acid metabolism | map00250 | Alanine, aspartate and glutamate metabolism | 1 |
| Metabolism | Lipid metabolism | map00140 | Steroid hormone biosynthesis | 4 |
| Metabolism | Carbohydrate metabolism | map00650 | Butanoate metabolism | 1 |
| Metabolism | Lipid metabolism | map00072 | Synthesis and degradation of ketone bodies | 1 |
| Metabolism | Lipid metabolism | map00564 | Glycerophospholipid metabolism | 1 |
| Metabolism | Xenobiotics biodegradation and metabolism | map00983 | Drug metabolism - other enzymes | 1 |
| Metabolism | Nucleotide metabolism | map00240 | Pyrimidine metabolism | 1 |
| Metabolism | Amino acid metabolism | map00280 | Valine, leucine and isoleucine degradation | 1 |
| Metabolism | Metabolism of cofactors and vitamins | map00760 | Nicotinate and nicotinamide metabolism | 1 |
| Metabolism | Lipid metabolism | map00100 | Steroid biosynthesis | 16 |
| Metabolism | Carbohydrate metabolism | map00020 | Citrate cycle (TCA cycle) | 2 |
| Metabolism | Lipid metabolism | map00590 | Arachidonic acid metabolism | 1 |
| Metabolism | Metabolism of terpenoids and polyketides | map00900 | Terpenoid backbone biosynthesis | 7 |
| Metabolism | Amino acid metabolism | map00220 | Arginine biosynthesis | 1 |
| Metabolism | Lipid metabolism | map00120 | Primary bile acid biosynthesis | 1 |
| Metabolism | Carbohydrate metabolism | map00620 | Pyruvate metabolism | 1 |

| | | | | |
|---|---|---|---|---|
| Metabolism | Metabolism of cofactors and vitamins | map00830 | Retinol metabolism | 1 |
| Metabolism | Glycan biosynthesis and metabolism | map00601 | Glycosphingolipid biosynthesis - lacto and neolacto series | 1 |
| Metabolism | Nucleotide metabolism | map00230 | Purine metabolism | 2 |
| Metabolism | Lipid metabolism | map01040 | Biosynthesis of unsaturated fatty acids | 1 |
| Metabolism | Carbohydrate metabolism | map00010 | Glycolysis / Gluconeogenesis | 1 |
| Genetic Information Processing | Folding, sorting and degradation | map04141 | Protein processing in endoplasmic reticulum | 2 |
| Genetic Information Processing | Replication and repair | map03030 | DNA replication | 2 |
| Genetic Information Processing | Transcription | map03040 | Spliceosome | 1 |
| Environmental Information Processing | Signaling molecules and interaction | map04080 | Neuroactive ligand-receptor interaction | 2 |
| Environmental Information Processing | Signal transduction | map04390 | Hippo signaling pathway | 1 |
| Environmental Information Processing | Signal transduction | map04010 | MAPK signaling pathway | 4 |
| Environmental Information Processing | Signal transduction | map04012 | ErbB signaling pathway | 1 |
| Environmental Information Processing | Signal transduction | map04310 | Wnt signaling pathway | 1 |
| Environmental Information Processing | Signal transduction | map04068 | FoxO signaling pathway | 2 |
| Environmental Information Processing | Signaling molecules and interaction | map04060 | Cytokine-cytokine receptor interaction | 2 |
| Environmental Information | Signal transduction | map04064 | NF-kappa B signaling pathway | 1 |

| | | | | |
|---|---|---|---|---|
| Processing | | | | |
| Environmental Information Processing | Signal transduction | map04151 | PI3K-Akt signaling pathway | 1 |
| Environmental Information Processing | Signal transduction | map04152 | AMPK signaling pathway | 3 |
| Environmental Information Processing | Signal transduction | map04350 | TGF-beta signaling pathway | 1 |
| Environmental Information Processing | Signal transduction | map04020 | Calcium signaling pathway | 3 |
| Cellular Processes | Transport and catabolism | map04146 | Peroxisome | 1 |
| Cellular Processes | Transport and catabolism | map04144 | Endocytosis | 2 |
| Cellular Processes | Cell growth and death | map04110 | Cell cycle | 4 |
| Cellular Processes | Cell motility | map04810 | Regulation of actin cytoskeleton | 1 |
| Organismal Systems | Endocrine system | map04920 | Adipocytokine signaling pathway | 1 |
| Organismal Systems | Immune system | map04621 | NOD-like receptor signaling pathway | 2 |
| Organismal Systems | Endocrine system | map04913 | Ovarian steroidogenesis | 3 |
| Organismal Systems | Immune system | map04640 | Hematopoietic cell lineage | 1 |
| Organismal Systems | Nervous system | map04726 | Serotonergic synapse | 1 |
| Organismal Systems | Immune system | map04659 | Th17 cell differentiation | 1 |
| Organismal Systems | Nervous system | map04725 | Cholinergic synapse | 1 |
| Organismal Systems | Endocrine system | map04910 | Insulin signaling pathway | 1 |
| Organismal Systems | Digestive system | map04972 | Pancreatic secretion | 1 |
| Organismal Systems | Digestive system | map04976 | Bile secretion | 2 |
| Organismal Systems | Digestive system | map04974 | Protein digestion and absorption | 1 |
| Organismal | Excretory system | map04964 | Proximal tubule | 1 |

| | | | | |
|---|---|---|---|---|
| Systems | | | bicarbonate reclamation | |
| Organismal Systems | Sensory system | map04750 | Inflammatory mediator regulation of TRP channels | 1 |
| Organismal Systems | Endocrine system | map03320 | PPAR signaling pathway | 3 |
| Organismal Systems | Endocrine system | map04922 | Glucagon signaling pathway | 1 |
| Organismal Systems | Development | map04380 | Osteoclast differentiation | 1 |
| Organismal Systems | Endocrine system | map04918 | Thyroid hormone synthesis | 1 |
| Organismal Systems | Endocrine system | map04912 | GnRH signaling pathway | 1 |
| Organismal Systems | Endocrine system | map04915 | Estrogen signaling pathway | 2 |
| Organismal Systems | Aging | map04213 | Longevity regulating pathway - multiple species | 1 |
| Organismal Systems | Aging | map04212 | Longevity regulating pathway - worm | 1 |
| Organismal Systems | Immune system | map04612 | Antigen processing and presentation | 1 |
| Human Diseases | Infectious diseases: Bacterial | map05120 | Epithelial cell signaling in Helicobacter pylori infection | 1 |
| Human Diseases | Infectious diseases: Parasitic | map05140 | Leishmaniasis | 1 |
| Human Diseases | Infectious diseases: Parasitic | map05142 | Chagas disease (American trypanosomiasis) | 1 |
| Human Diseases | Infectious diseases: Parasitic | map05145 | Toxoplasmosis | 2 |
| Human Diseases | Immune diseases | map05321 | Inflammatory bowel disease (IBD) | 1 |
| Human Diseases | Infectious diseases: Bacterial | map05134 | Legionellosis | 1 |
| Human Diseases | Substance dependence | map05034 | Alcoholism | 1 |
| Human Diseases | Cancers: Specific types | map05220 | Chronic myeloid leukemia | 1 |
| Human Diseases | Infectious diseases: Bacterial | map05152 | Tuberculosis | 1 |
| Human Diseases | Infectious diseases: | map05144 | Malaria | 1 |

| | | | | |
|---|---|---|---|---|
| | Parasitic | | | |
| Human Diseases | Cardiovascular diseases | map05418 | Fluid shear stress and atherosclerosis | 2 |
| Human Diseases | Cardiovascular diseases | map05410 | Hypertrophic cardiomyopathy (HCM) | 1 |
| Human Diseases | Neurodegenerative diseases | map05020 | Prion diseases | 2 |
| Human Diseases | Infectious diseases: Viral | map05166 | HTLV-I infection | 6 |
| Human Diseases | Infectious diseases: Viral | map05164 | Influenza A | 4 |
| Human Diseases | Infectious diseases: Viral | map05162 | Measles | 1 |
| Human Diseases | Infectious diseases: Viral | map05161 | Hepatitis B | 1 |
| Human Diseases | Infectious diseases: Viral | map05169 | Epstein-Barr virus infection | 1 |
| Human Diseases | Cardiovascular diseases | map05414 | Dilated cardiomyopathy | 1 |
| Human Diseases | Drug resistance: Antineoplastic | map01522 | Endocrine resistance | 1 |
| Human Diseases | Cancers: Specific types | map05210 | Colorectal cancer | 1 |
| Human Diseases | Cancers: Specific types | map05212 | Pancreatic cancer | 1 |
| Human Diseases | Cancers: Specific types | map05219 | Bladder cancer | 1 |
| Human Diseases | Cancers: Specific types | map05211 | Renal cell carcinoma | 1 |
| Human Diseases | Infectious diseases: Parasitic | map05146 | Amoebiasis | 2 |
| Human Diseases | Cancers: Overview | map05205 | Proteoglycans in cancer | 1 |
| Human Diseases | Cancers: Overview | map05203 | Viral carcinogenesis | 1 |
| Human Diseases | Cancers: Overview | map05200 | Pathways in cancer | 1 |
| Human Diseases | Immune diseases | map05322 | Systemic lupus erythematosus | 1 |
| Human Diseases | Immune diseases | map05323 | Rheumatoid arthritis | 1 |
| Human Diseases | Endocrine and metabolic diseases | map04933 | AGE-RAGE signaling pathway in diabetic complications | 3 |
| Human Diseases | Endocrine and metabolic diseases | map04931 | Insulin resistance | 1 |

**Table S8** Significantly up/down-regulated pathways with their proteins.

| pathway | *p*-value | gene name | description | up/down |
|---|---|---|---|---|
| **Lipid metabolism** | | | | |
| Steroid biosynthesis | $2.85x10^{-9}$ | DHCR7 | 7-dehydrocholesterol reductase | up |
| | | TM7SF2 | delta14-sterol reductase | up |
| | | SC5DL | delta7-sterol 5-desaturase | up |
| | | SQLE | squalene monooxygenase | up |
| | | FDFT1 | farnesyl-diphosphate farnesyltransferase | up |
| | | EBP | cholestenol Delta-isomerase | up |
| | | LSS | lanosterol synthase | up |
| | | CYP51 | sterol 14alpha-demethylase | up |
| | | NSDHL | sterol-4alpha-carboxylate 3-dehydrogenase (decarboxylating) | up |
| | | MESO1 | methylsterol monooxygenase | up |
| | | DHCR24 | delta24-sterol reductase | up |
| | | HSD17B7 | 17 beta-estradiol 17-dehydrogenase / 3 beta-hydroxysteroid 3-dehydrogenase | up |
| Steroid hormone biosynthesis | 0.005 | CYP7A1 | cholesterol 7-alpha-monooxygenase | down |
| | | HSD17B7 | hydroxysteroid (17-beta) dehydrogenase 7 | up |
| **Metabolism of terpenoids and polyketides** | | | | |
| Terpenoid backbone biosynthesis | $4.96x10^{-9}$ | HMGCS1/HMGCR | 3-hydroxy-3-methylglutaryl-CoA synthase 1 | up |
| | | PMVK | phosphomevalonate kinase | up |
| | | FDPS | farnesyl diphosphate synthase | up |
| | | MVD | mevalonate diphosphate decarboxylase | up |
| | | IDI1 | isopentenyl-diphosphate delta isomerase 1 | up |

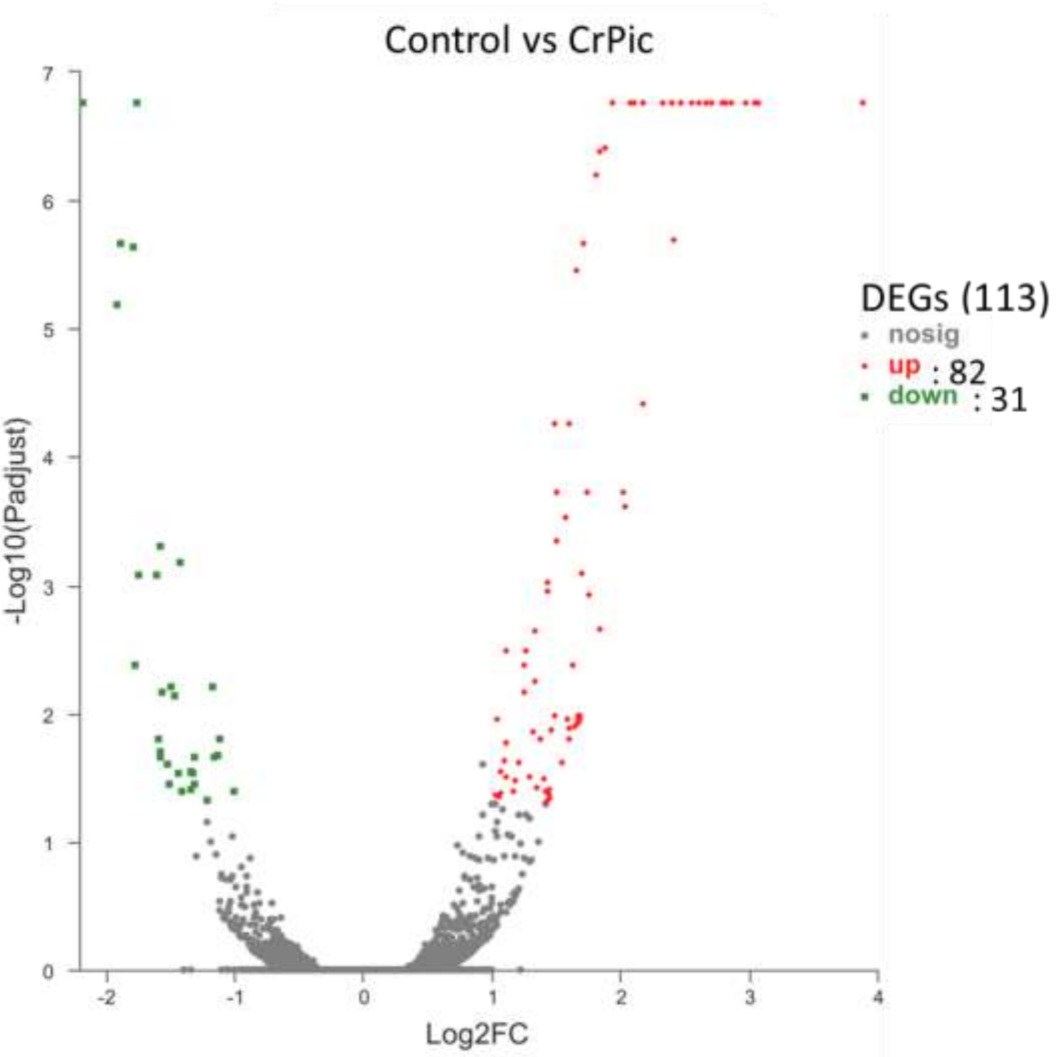


**Fig. S1 The volcano plot of the gene expression differences between the Control and CrPic group**

Each point represents one gene that is detectable in both groups. Red dots and green dots indicate the significantly up-regulated and down-regulated genes, respectively. Black dots show genes with no significant difference in expression between the Control and CrPic group. The horizontal axis represented the log-ratio (gene expression fold change in different samples) and the vertical axis represented the probability for each gene of being differentially expressed. The total DEGs number identified by FDR-adjusted $p$-value < 0.05 was shown.

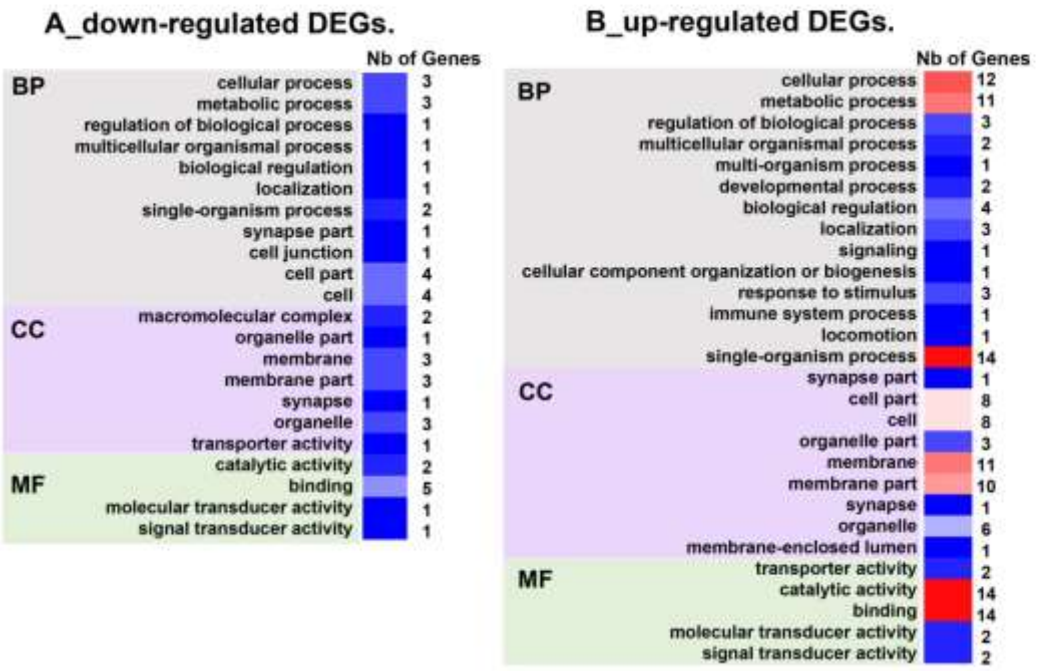


**Fig. S2 The heat map analysis of differentially expressed genes (DEGs) using Gene Ontology (GO) terms annotation cluster**

The left part (A) represented down-regulated genes, and the right part (B) represented up-regulated genes. The color bar from blue to red represented that the number of genes increased from left to right. The GO terms were enriched as biological process (BP, grey), Cellular components (CC, purple), or molecular function (MF, light green).

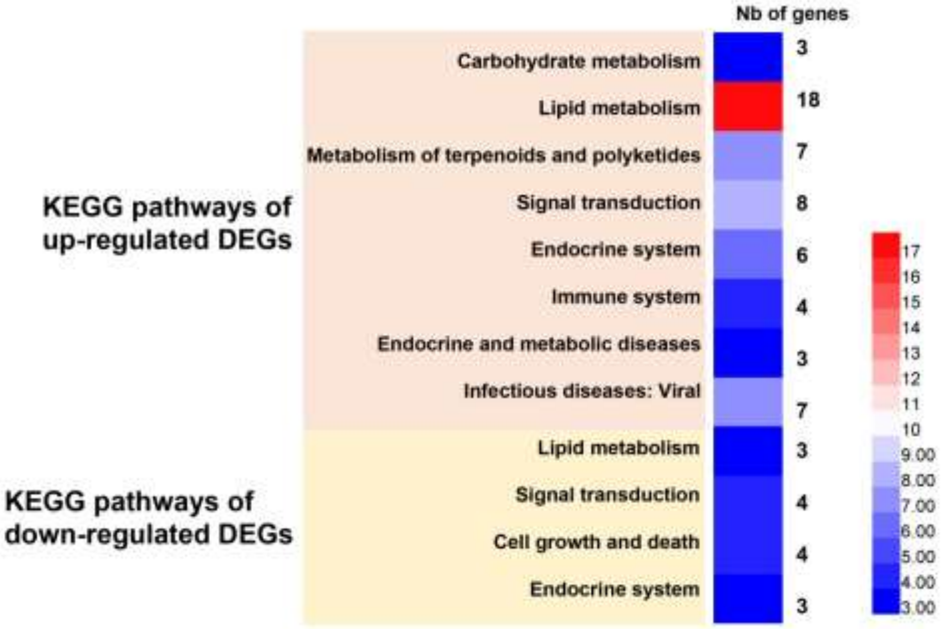


**Fig. S3 The heat map analysis of 113 differentially expressed genes (DEGs) using Kyoto Encyclopedia of Genes and Genomes (KEGG) pathway**

The left axis was the name of the KEGG metabolic pathway, and the right axis was the number of the differentially expressed genes (DEGs) annotated in the pathway. The upper part represented KEGG pathways of up-regulated genes (pink), and the bottom part represented KEGG pathways of down-regulated genes (light orange). The color bar from blue to red represented that the number of genes increased from down to up.

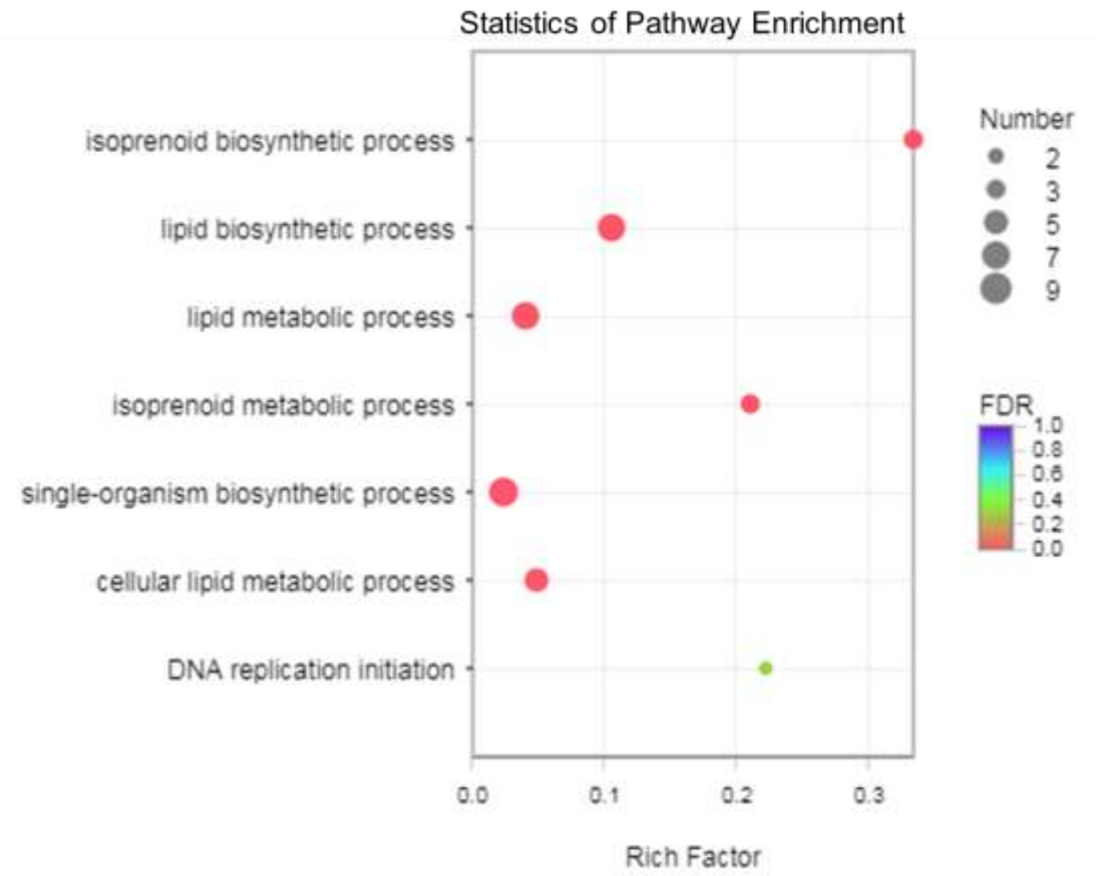


**Fig. S4 The scatter plot of Gene Ontology (GO) enrichment among the 113 differentially expressed genes (DEGs)**

The vertical axis represented the pathway categories, and the horizontal axis shows the rich factor. The greater the rich factor, the greater the degree of enrichment. The point size shows the number of DEGs among the pathway. The bigger the point size, the more gene numbers in the pathway. The point color shows different Q values (FDR) as indicated on the right.

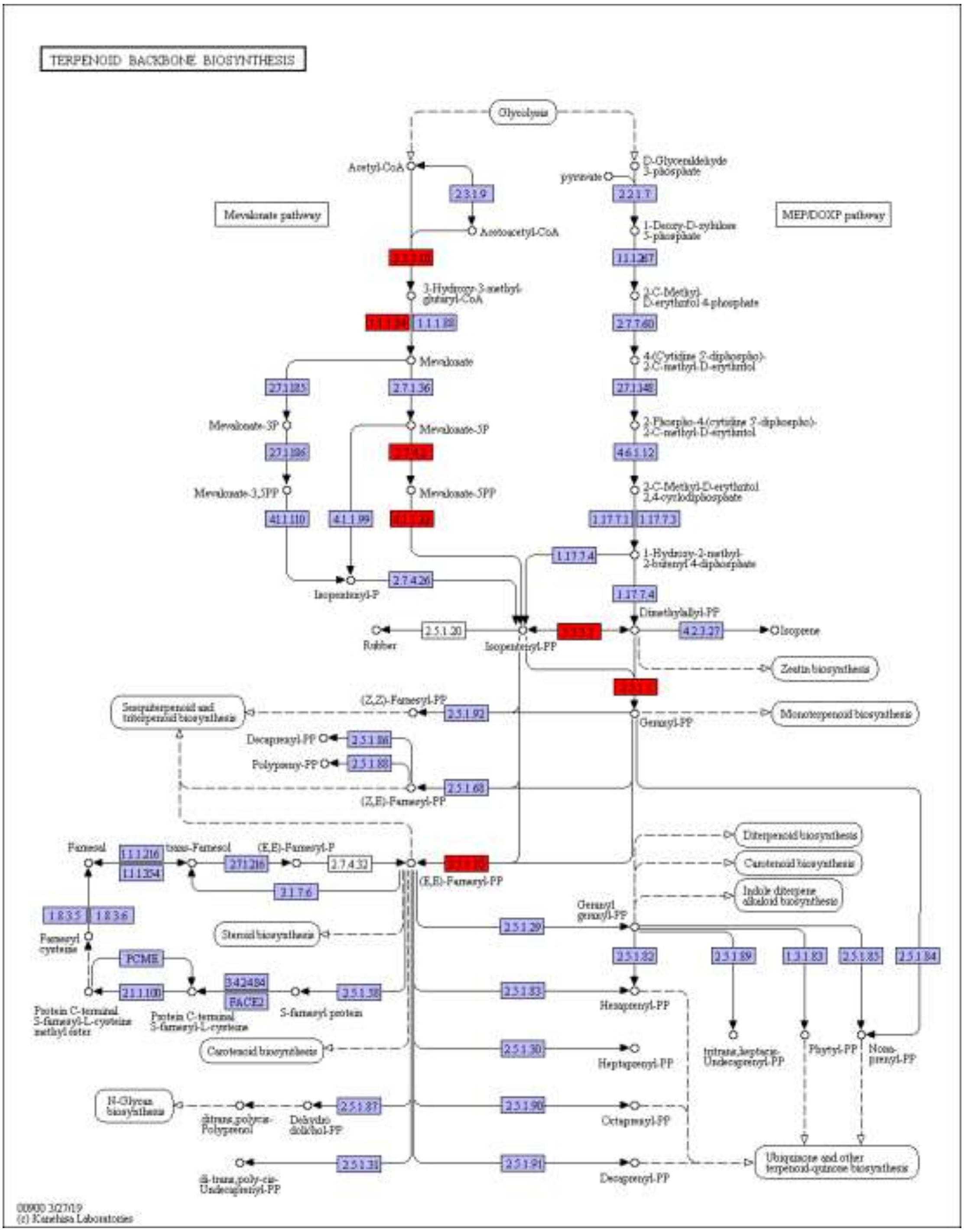


**Fig. S5 terpenoid backbone biosynthesis pathway.**

The red boxes represented significantly up-regulated genes involved in the pathway. The expression levels of genes encoding hydroxy-3-methylglutaryl-CoA reductase (*hmgcr*), phosphomevalonate kinase (*pmvk*), diphosphomevalonate decarboxylase (*mvd*), isopentenyl-diphosphate delta-isomerase (*idi*), farnesyl diphosphate synthease (*fdps*), all of which were increased significantly under CrPic treatment.

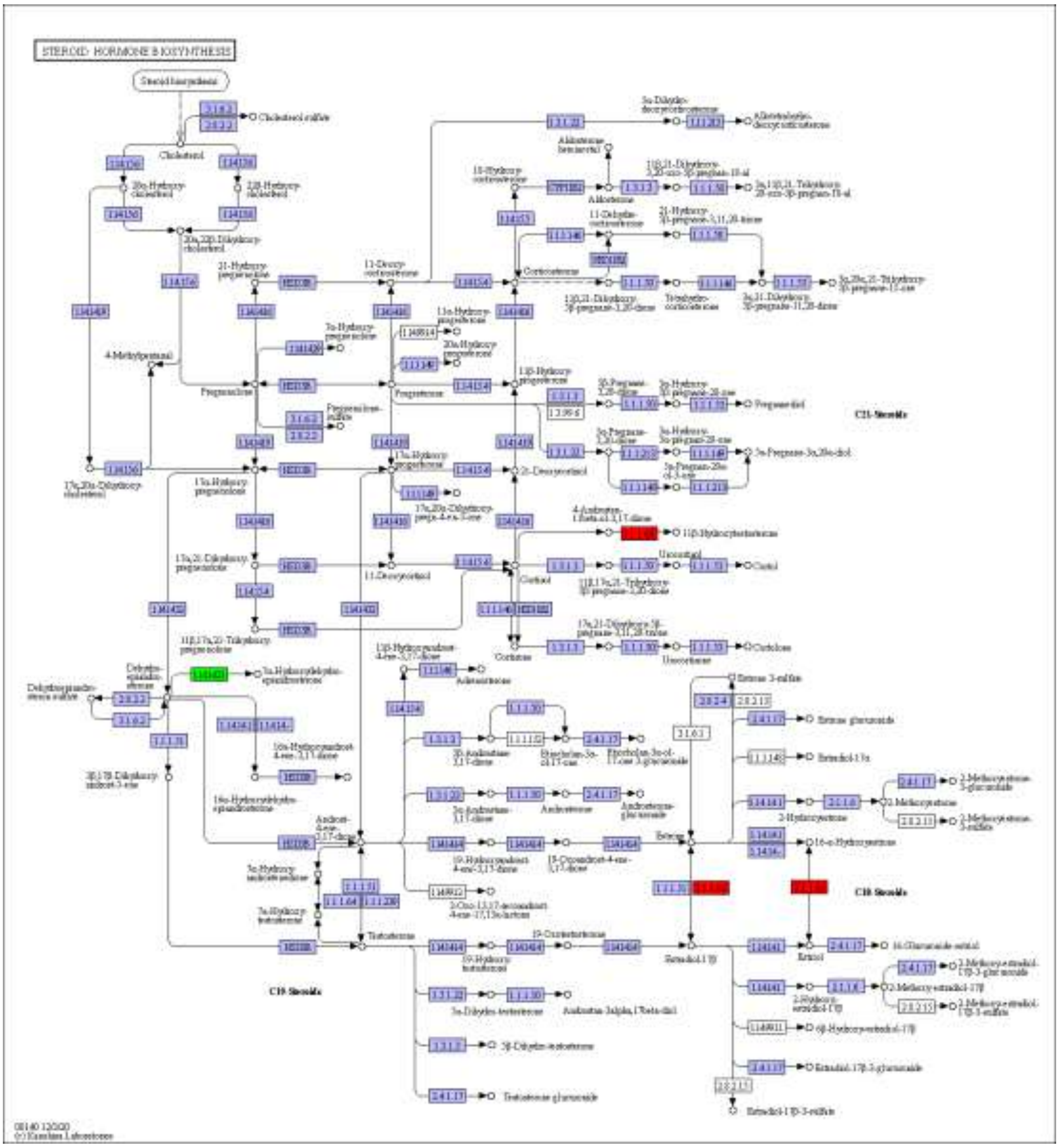


**Fig. S6 steroid hormone biosynthesis pathway.**

The boxes represented the significantly up-regulated (red box) and down-regulated (green box) genes involved in the pathway. The expression levels of genes encoding hydroxysteroid 17-beta dehydrogenase 7 (*hsd17b7*) was increased significantly under CrPic treatment. The expression levels of genes encoding cholesterol 7alpha-monooxygenase (*cyp7a1*) was decreased significantly under CrPic treatment.
